# Primary care physicians’ knowledge and attitudes about obesity, adherence to treatment guidelines and association with confidence to treat obesity: a Swedish survey study

**DOI:** 10.1186/s12875-022-01811-x

**Published:** 2022-08-16

**Authors:** Daniel Carrasco, Hans Thulesius, Ulf Jakobsson, Ensieh Memarian

**Affiliations:** 1 Department of Clinical Sciences in Malmö, Lund University, Internal Medicine Research Group, Jan Waldenströms gata 15, 5th floor, Skåne University Hospital, S-20502 Malmö, Sweden; 2 Department of Research and Development, Region Kronoberg, Växjö, Sweden; 3 Department of Clinical Sciences Malmö, Faculty of Medicine, Lund University, Malmö, Sweden; 4 Faculty of Health and Life Sciences, Department of Medicine and Optometry, Linnaeus University, Kalmar, Sweden; 5 Center for Primary Health Care Research, Department of Clinical Sciences in Malmö, Lund University, Malmö, Sweden

**Keywords:** Obesity, Survey study, Primary care physician, Knowledge, Attitudes, Obesity treatment

## Abstract

**Background:**

Obesity is a chronic disease with increasing prevalence. We aimed to explore primary care physicians’ knowledge and attitudes about obesity and how knowledge and attitudes are associated with confidence and adherence to obesity guidelines and barriers to obesity treatment.

**Methods:**

A questionnaire survey was sent by e-mail to 1642 primary care physicians in four regions in Sweden. The survey focused on the physicians’ knowledge, attitudes towards obesity, confidence in obesity management, adherence to obesity guidelines and barriers to optimal care. We created different statistical indices for knowledge, attitudes and adherence. To analyse the correlation between these indices, we used linear regression analyses.

**Results:**

Replies from 235 primary care physicians yielded a response rate of 14.3%. Most physicians answered correctly that obesity is a disease (91%), that obesity regulation sits in the hypothalamus (70%) and that obesity is due to disorders of appetite regulation (69%). However, 44% of the physicians thought that the most effective weight reduction method for severe obesity was lifestyle changes; 47% believed that obesity is due to lack of self-control, 14% mentioned lack of motivation and 22% stated laziness. Although 97% believed that physicians can help individuals with obesity and 56% suggested that obesity treatment should be prioritised, 87% of the physicians expressed that losing weight is the patients’ responsibility. There was a positive association between higher knowledge and better adherence to obesity guidelines (B = 0.07, CI 0.02–0.12, p-value = 0.005) and feeling confident to suggest medication (p < 0.001) or bariatric surgery (p = 0.002). While 99% of the physicians felt confident to discuss lifestyle changes, 67% and 81% were confident to suggest medication or bariatric surgery, respectively. Respondents perceived that the greatest barrier in obesity management was lack of time (69%) and resources (49%).

**Conclusion:**

There was a positive association between Swedish primary care physicians’ knowledge and adherence to obesity guidelines and being more confident to suggest obesity treatment. Yet, many physicians had an ambivalent attitude towards obesity management.

**Supplementary Information:**

The online version contains supplementary material available at 10.1186/s12875-022-01811-x.

## Background

The prevalence of obesity is increasing around the world and it is one of the major global health problems [1]. According to the Public Health Agency of Sweden, the proportion of the population aged 16–84 that are either overweight or obese has increased from 46 to 52% between 2006 and 2020 and obesity is considered to be one of the five main risk factors in Sweden that cause loss of healthy years of life [2].

Body mass index (BMI) is used to define overweight and obesity in adults. According to the World Health Organization (WHO) classification, an adult that has a BMI 25–29.99 kg/m^2^ is classified as being overweight, BMI 30–39.99 kg/m^2^ is obese class I and II and BMI > 40 kg/m^2^ is classified as obese class III/severely obese.

Obesity is a chronic disease and is a risk factor for serious comorbidities such as diabetes type 2, hypertension, obstructive sleep apnoea, cardiovascular disease, liver disease, dyslipidaemia, joint pain, several types of cancer, depression and infertility [3–9]. Obesity [9] is highly associated with increased mortality, lower quality of life, social stigmatisation, discrimination at an individual, micro level [10], and increased costs for the society and health care system at a macro level [7].

There are numerous interventions that can be recommended for individuals with obesity, i.e. dietary changes, physical activity, behavioural changes, pharmacological treatment, and bariatric surgery.

In Sweden, most individuals with obesity primarily see a primary care physician when they need help in treating obesity. There are different criteria for various approved treatment options based on BMI and other obesity related comorbidities with 4 levels of treatment for obesity:

1. *Lifestyle intervention in primary health care*: Individuals with BMI > 25 kg/m^2^ may consult a nurse or dietician specially trained in lifestyle changes.
2. *Medication*: Xenical® (Orlistat), Saxenda® (Liraglutid) and Mysimba® (Bupropion and Naltrexon) could be prescribed to individuals with BMI ≥ 30 kg/m^2^ (or BMI ≥ 28 kg/m^2^ in addition to other obesity related comorbidities) in Sweden June 2022.
3. *Referral to obesity clinic*: Individuals with BMI > 35 kg/m^2^ or BMI > 30 kg/m^2^ in addition to obesity related comorbidities and unsatisfactory effect of earlier lifestyle modification, with at least one attempt through organised intervention at primary health care may be referred to a clinic for patients with obesity, offering group therapy or individual therapy in combination with very low-calorie diet (VLCD) or medication.
4. *Bariatric surgery*: According to national criteria in Sweden, individuals who are refractory to nonsurgical treatment and have a BMI ≥ 40 kg/m^2^ or a BMI ≥ 35 kg/m^2^ in addition to an obesity-related comorbidity, are eligible to undergo bariatric surgery [11.

There are multiple barriers which might influence the effective management of obesity. Although obesity is a disease, it is not always treated accordingly, with discrepancies in perceptions and attitudes toward obesity as a potential barrier to obesity management [12]. One barrier might be that primary care physicians and other healthcare professionals lack knowledge [13] and training in the behavioural counselling that is necessary for lifestyle interventions. In a qualitative study from the USA, physicians acknowledged that they had limited knowledge about obesity treatment options [14]. In an earlier study, we found a significant positive association between primary care physicians’ knowledge and positive attitudes about obesity and willingness to refer patients to bariatric surgery [15]. Despite increasing awareness about the pathophysiological basis of obesity such as hereditary factors, a strong stigma and negative attitudes persists towards individuals with obesity even among health care providers [16] which might discourage choosing effective evidence-based interventions. Low prioritization and lack of follow-up care are other barriers to effective obesity management [12].

Despite the increasing prevalence of obesity and its association with other comorbidities, physicians’ rates of diagnosing and management of obesity are low. In a cross-sectional analysis of 696 million ambulatory care visits in the USA, 70% of patients with obesity were not diagnosed, and 63% received no consultation about lifestyle changes or weight reduction [17]. In another cross-sectional study by Bleich et al., in ambulatory care (*N* = 2458), only 28.9% of adults with obesity received an obesity diagnosis and only 17.6% received counselling for weight reduction [18].

The primary aim of this study was to explore the *attitudes* of primary care physicians towards obesity and their *knowledge* about available treatment options. The secondary aim was to investigate primary care physicians’ *adherence* to obesity guidelines in the management of adult obese patients and its association with their attitudes and knowledge about obesity. The third aim was to identify *barriers* that prevent primary care physicians from adhering to obesity guidelines. We hypothesised that low knowledge about obesity and its treatment options among primary care physicians would correlate to negative attitudes about obesity and lower adherence to obesity treatment guidelines.

## Methods

 In 2021, a questionnaire survey was sent by e-mail to 1392 primary care physicians in four regions in Sweden: Stockholm (*N* = 41), Skåne (*N* = 1100), Västra Götaland (*N* = 171), Kronoberg (*N* = 80) and to 250 primary care physicians who were members of the e-mail-list ORDBYTE, which is a platform for the exchange of ideas and experiences among general practitioners in Sweden since 1999. We selected the above regions since the first three are the most populated regions in Sweden. Kronoberg represents a 2% cross section of Sweden both regarding population demographics and surface area. Three of the authors worked in the Skåne region and had access to e-mail addresses through the regional councils. For Stockholm and Västra Götaland the head of primary care clinics were asked to participate and those clinics that agreed to participate received a link to the questionnaire that was later distributed to primary care physicians.

The primary care physicians who received the questionnaire were briefly informed about obesity and the study objectives. The participation was voluntary, responses were collected anonymously and could not be traced back to any of the respondents. The potential participants were also informed that they could terminate their participation at any time. A survey response by the primary care physicians was considered as consent to participate. According to Swedish legislation (law nr 2003:460), an application to the Ethical review Authority was not required, since the survey was anonymous and did not contain sensitive data. Prior to the start of the study the Ethical Review Authority in Sweden was contacted which confirmed that the study did not require application to EPM.

The survey consisted of two parts:

1. Items about attitudes and knowledge regarding obesity, confidence in obesity management, adherence to obesity treatment guidelines, barriers to optimal care and respondent demographics.
2. Clinical case vignettes describing patients with obesity. Questions were designed to explore how respondents would manage the patients described in the vignettes.

The electronic questionnaire was designed taking the following steps:

1. We, the authors, discussed factors that could affect physicians’ decision-making regarding obesity management. The discussion was based on our own experiences. Items regarding barriers were developed with input from a group of physician colleagues in primary care.
2. We searched the literature in order to find questionnaire surveys with one or more items of interest for our study and we found nine studies [14, 19–26]. Additional items were created. The survey included 35 items whereof 11 items and three modified versions of case vignettes came from the literature (items 6–12, 16, 21–23, 30 and items related to case vignettes). The items focused on knowledge about obesity (e.g. BMI criteria for different treatment options) and attitudes towards obesity (e.g. obesity is due to poor self-control), adherence to obesity treatment guidelines (e.g. suggestion about a special treatment), barriers (e.g. lack of time) and feeling confident to treat individual with obesity (e.g. with obesity medication). Most of the items were graded on a 5-point Likert scale: 1 = strongly disagree; 2 = disagree; 3 = neither agree nor disagree; 4 = agree; 5 = strongly agree and 6 = do not know. Apart from these obesity focused items the questionnaire contained demographic information such as age, gender, medical specialisation, years of physician experience, and if the respondents worked part-time or full-time and if they worked at a private or public clinic.
3. Items from other questionnaire surveys were translated first from English to Swedish and consecutively from Swedish back to English by two different researchers highly skilled in both languages to ensure that the meaning of the items did not change after the translation.
4. Ten primary care physicians were invited to respond to the first version of the questionnaire and give comments in order to assess the content and the face validity of the survey.
5. We modified the survey based on the received comments that were relevant for the purpose of the study. The last version of the questionnaire contained 35 items.
6. The questionnaire was produced electronically in REDCap®, a web application for handling electronic questionnaires.
7. In order to check if the survey link worked appropriately, we sent the survey electronically to the authors and 10 primary care physician colleagues at our clinic. The survey results from these 10 colleagues were not included in the final survey.

After the above steps, the questionnaire was sent out to physicians with available email addresses, who worked in primary care in Stockholm, Västra Götaland, Skåne, Kronoberg regions in Sweden or were members of ORDBYTE. Some of the members of ORDBYTE worked in the four regions. Therefore, prior to clicking “send”, we did a double check of the ORDBYTE e-mail addresses. Some participants may have received the questionnaire survey twice, and they were asked to answer the questionnaire only once. The survey was sent out for the first time in March 2021. We sent out two reminders, one in April and one in May 2021. The data collection lasted for three months.

We analysed the data using descriptive statistics and regression models. We also developed indices for items regarding knowledge about obesity (items 13, some of the options of item 17, 18 to 20, 22 to 29), attitudes about obesity (items 7 to 10, 12 and options 8 and 9 of item 17) and adherence to obesity treatment guidelines (items 11, 31 to 34). A detailed description of how the indices were developed is as follows:

Item 20 was coded in accordance with a Likert scale, while items 7 to 12 were coded with yes/no options. The rest of the items were multiple choice questions. The index score for *knowledge* comes from items 13, 17 (option 1 to 7 and 10) to 20, 22 to 29, where the respondents could get 1–2 points for each item. High points were given if the physicians answered correctly to these items. If the physicians had chosen options one or two for item 18, they received one point, for option three they received two points and zero points for option four. For item 20, if the respondents had chosen options four or five for each part, they received one point, otherwise zero points.

The index score for *attitudes* comes from items 7 to 10, 12, 17 (options 8 and 9). For each item or option, the physicians could get one point. For example, higher scores for items about attitudes generated a higher index score for positive attitudes.

The index score for *adherence* to obesity treatment guidelines comes from items 11 and case vignettes 1 to 3 (items 31 to 34). The respondents could get one point for a right answer for items 11, 31, 32 and 34. For item 33, the respondents could get 0 to 3 points. The English version of the questionnaire is attached as an Additional file 1: Appendix 1. Linear regression models were used to assess the associations between the indices. We used the SPSS Statistics, version 25 for statistical analyses and p < 0.05 was considered statistically significant.

## Results

### Demographic data

 The questionnaire survey was sent out to 1392 primary care physicians in Skåne, Stockholm, Västra Götaland, Kronoberg regions and 250 members of ORDBYTE. We received 175 responses from the above four regions and 60 responses from ORDBYTE's members, i.e. a total of 235 primary care physicians out of 1642 replied yielding a response rate of 14.3%.

A total of 23 questionnaires were not complete and had internal missing answers for 1 to 3 items (15 from the four regions and eight from ORDBYTE). Of the 235 respondents, 44% (*n* = 103) were men and 56% (*n* = 131) women (1 missing answer). Most of the respondents (73%) were specialists in general practice and 94 (56%) of them had worked for more than 10 years in primary care. Most of the respondents (65%) worked in public primary care and 34% worked in private primary care, see Table 1.

**Table 1:** Demographic data of the primary care physician respondents

	Men, nr (%)	Women, nr (%)	Total, nr (%)
**Number of participants**			235
**Gender** ^**1**^	103 (44%)	131 (56%)	234 (99.6%)
**Age** ^**2**^			
• **Age < 35 year**	14 (14%)	13 (10%)	27 (11%)
• **Age 35–49 year**	38 (37%)	68 (52%)	107 (46%)
• **Age > 49 year**	50 (50%)	50 (38%)	100 (43%)
**Speciality** ^3^			232 (99%)
• **Specialist in general practice**	74 (73%)	95 (73%)	169 (73%)
• **Primary care physician not specialist in general practice**	27 (27%)	35 (27%)	62 (27%)
**Speciality experience** ^4^			
• **Worked as specialist in general practice 0–5 year**	20 (27%)	29 (30%)	49 (29%)
• **Worked as specialist in general practice 6–10 year**	9 (12%)	16 (17%)	25 (15%)
• **Worked as specialist in general practice > 10 year**	44 (60%)	50 (53%)	94 (56%)
**Full-time/part-time** ^5^			
• **Working full-time**	53 (52%)	39 (30%)	92 (39%)
• **Working part-time**	49 (48%)	91 (70%)	141 (61%)
**Public/private clinic** ^6^			
• **Public primary health care centre**	67 (66%)	85 (65%)	153 (66%)
• **Private primary health care centre**	34 (34%)	45 (35%)	79 (34%)

Item 1–6

^1^Missing = 1

^2^Missing = 2

^3^Missing = 3

^4^Missing = 1

^5^Missing = 2.

^6^Missing = 3

**Fig. 1:**
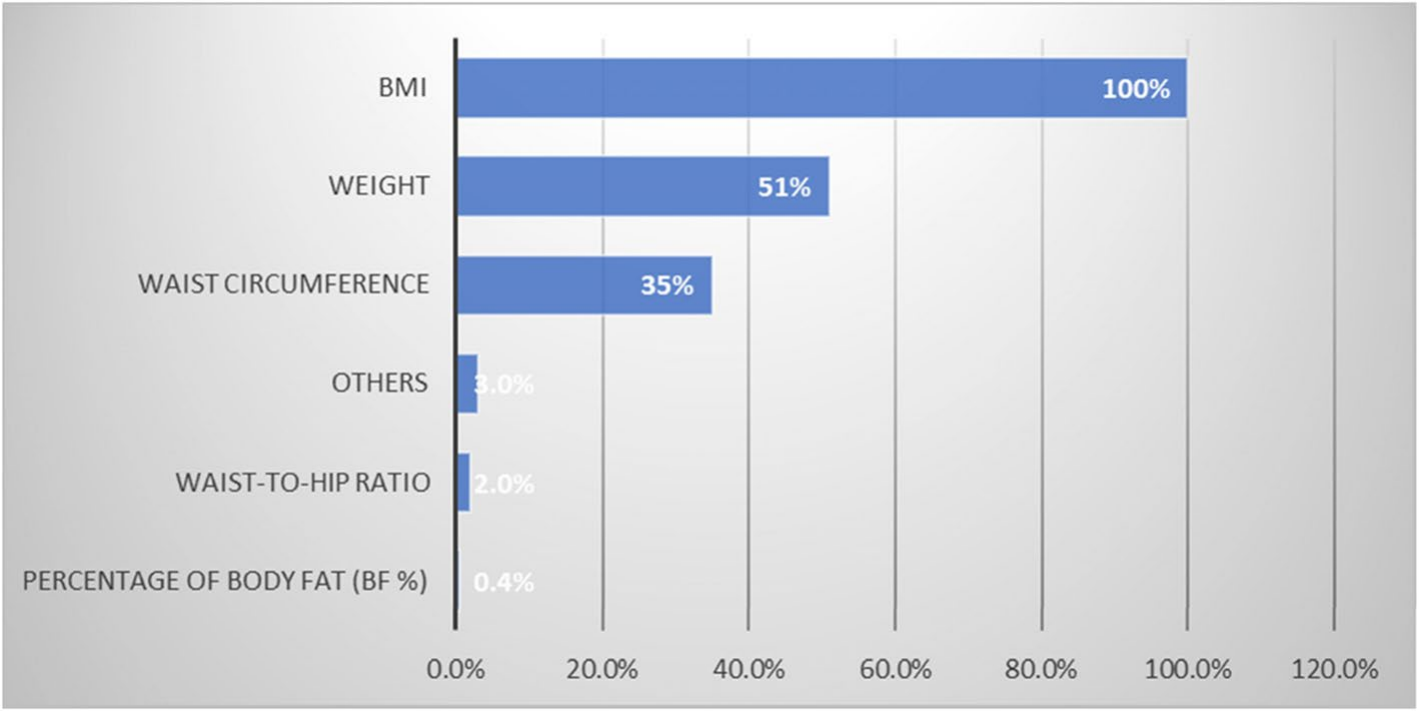
Which of the measures do you use at your clinic to screen for obesity? (Item 15)

BMI was by far the most common measure used by the primary care physicians to screen for obesity (100%), Fig. 1.

**Fig. 2:**
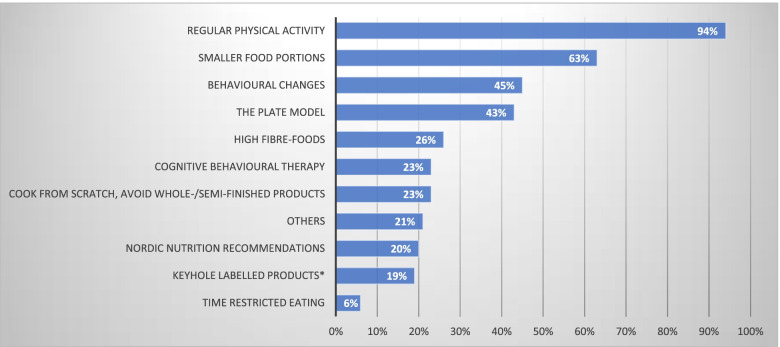
Which lifestyle changes do you usually suggest to your patients with obesity? (Item 16). *Food labelled with the Keyhole symbol has a healthier fat composition, contains less sugars and salt, more dietary fibre and wholegrain

The most common lifestyle changes recommended by primary care physicians to their patients with obesity was regular physical activity (94%) and smaller food portions (63%) Fig. 2.

### Knowledge about obesity

A large majority of the respondents (*n* = 214, 91%) answered correctly that obesity is a disease (item 13). The proportion of primary care physicians that stated various causes of obesity was as follows: genetics = 94%, changes in the society (e.g. fast food industry, abundance of cheap food) = 94%, socioeconomic status = 91%, lifestyle changes = 93%, pregnancy = 51%, obesogenic medication = 89%, intestine microbiota = 29% and psychological disorders = 91%.

A majority of the respondents (*n* = 164, 70%) answered correctly that hunger and satiety regulation sits in the hypothalamus and not in the brain cortex, and 69% (*n* = 163) believed that obesity is due to appetite regulation disorders, i.e. constantly feeling hungry or late satiety. However, 44% of the physicians perceived that lifestyle changes were the most effective weight reduction method for individuals with severe obesity and 45% expressed that more than 20% of individuals with severe obesity achieved a sustained 10% weight reduction after five years with lifestyle changes.

More than half of the respondents (*n* = 137, 58%) did not believe that obesity treatment medications are an effective way to lose weight for individuals with moderate obesity (BMI 30 to 39.9 kg/m^2^) and 20% (*n* = 47) were uncertain (Item 21).

### Attitudes about obesity

Almost half of the respondents, (*n* = 111, 47%), believed that one of the causes of obesity is lack of self-control (item 17, option 8) and 22% (*n* = 52) perceived that obesity is due to laziness (item 17, option 9) and 14% (*n* = 32) responded that individuals with obesity lack motivation to lose weight.

Although 97% of the respondents (*n* = 227) believed that physicians are able to help individuals with obesity to achieve a healthier weight (item 7) and 56% (*n* = 132) suggested that treatment of obesity should be prioritised (item 12), nevertheless 87% (*n* = 204) of the physicians expressed that losing weight is primarily the patients’ responsibility (item 9).

### Adherence to obesity treatment guidelines

A majority of the respondents (*n* = 191, 81%) claimed that they suggested obesity treatment to their patients when they were consulted for obesity related comorbidities (item 11). This was confirmed by the responses to item 31, which was a clinical case vignette, and 84% (*n* = 189) responded that there is a great probability that they discuss weight reduction with the patients who had an obesity comorbidity. In response to case vignette item 32, 42% (*n* = 98) of physicians expressed willingness to refer a patient who was eligible for bariatric surgery to an obesity clinic for operation. Regarding the same patient, only 3% (*n* = 7) believed that no further investigation of the patient’s clinical status (such as blood sugar, lipids and obstructive sleep apnoea) was needed (item 33). For the last case vignette (item 34), a patient with obesity (BMI = 38 kg/m^2^) and several obesity related comorbidities and family history of cardiovascular mortality, 43% (*n* = 102) of the physicians were willing to refer the patient for bariatric surgery.

### Knowledge and attitudes to obesity and their association with respondents’ characteristics and adherence to obesity treatment guidelines

We created different statistical indices for items regarding knowledge, attitudes to obesity and adherence to obesity guidelines to be able to analyse the association between these items. Median value for knowledge index was 19 out of a total of 28, i.e. half (50%) of the respondents answered correctly to more than 68% of the items regarding knowledge. Detailed information about these indices is presented in Table 2.

**Table 2:** Description of indices for knowledge, attitudes about obesity and adherence to obesity treatment guidelines

Indices	Number (N)	Mean value^a^	Median^b, ^(%)^c^	Respondents’ min-max	Theoretical range	Standard deviation
Knowledge^1^	235	18.9	19 (68%)	6–26	0–28	3.6
Attitudes^2^	232^4^	4.5	5 (62%)	0–7	0–8	1.4
Adherence to guidelines^3^	232^5^	5.7	6 (75%)	2–8	0–8	1.3

^a^The sum of the values divided by the number of values

^b^The value separating the higher half from the lower half of the data sample

^c^The median value divided by total result in percentage

^1^Items 13, 17 (options 1–7 and 9), 18–20, 22–29

^2^Items 7–10, 12, 17 (options 8–9)

^3^Items 11, 31–34

^4^Missing=3

^5^Missing=3

In Table 3, in univariate and multiple models, the association between respondents’ knowledge and attitudes to obesity with their characteristics and adherence to obesity treatment guidelines are presented using a linear regression. There was a positive association between having higher knowledge about obesity and better adherence to obesity guidelines (B = 0.07, CI 0.02 to 0.12 in multiple model, p-value = 0.005). Female gender was associated with higher knowledge (B = 1.43, CI 0.50 to 2.37 in multiple model, p-value = 0.003) and better attitudes to obesity (B = 0.65, CI 0.27 to 1.03 in multiple model, p- value = 0.001 ). Younger physicians (age < 35 years) had generally higher knowledge about obesity (B = 2.96, CI 0.97 to 4.94 in multiple model, p-value = 0.004). Those physicians who worked part-time had higher knowledge (B = 1.16, CI 0.19 to 2.12 in multiple model, p-value = 0.020) and better attitudes to obesity (B = 0.40, CI 0.04 to 0.77 in univariate model, p-value = 0.031).

**Table 3:** Linear regression: Association between knowledge, attitude and adherence to obesity guidelines and individual respondents’ characteristics

	Knowledge				Attitudes				Adherence			
	Univariate model		Multiple model^**^		Univariate model		Multiple model^**^		Univariate model		Multiple model^**^	
	p-value*	B (95% CI)	p-value	B (95% CI)	p- value^*^	B (95% CI)	p-value^*^	B (95% CI)	p-value^*^	B (95% CI)	p-value^*^	B (95% CI)
Knowledge					0.170	0.04 (-0.02 to 0.09)	0.81	0.01 (-0.05 to 0.06)	**0.008**	0.06 (0.02 to 0.11)	**0.005**	0.07 (0.02 to 0.12)
Attitudes									0.844	-0.01 (-0.14 to 0.11)	0.694	-0.03 (-0.16 to 0.10)
Adherence												
Gender (1 = man, 2 = woman)	**< 0.005**	1.63 (0.72 to 2.54)	**0.003**	1.43 (0.50 to 2.37)	**0.001**	0.62 (0.27 to 0.98)	**0.001**	0.65 (0.27 to 1.03)	0.190	0.23 (-0.11 to 0.57)	0.234	0.23 (-0.15 to 0.60)
Age												
• < 35 years	**0.002**	2.42 (0.91 to 3.93)	**0.004**	2.96 (0.97 to 4.94)	0.469	-0.22 (-0.81 to 0.38)	0.837	0.06 (-0.70 to 0.86)	0.734	0.10 (-0.47 to 0.66)	0.632	-0.19 (-0.95 to 0.58)
• 35–49 years	0.132	0.74 (-0.23 to 1.71)	0.170	0.91 (-0.39 to 2.21)	0.427	-0.15 (-0.54 to 0.23)	0.747	-0.09 (-0.60 to 0.43)	0.365	-0.17 (-0.53 to 0.20)	0.084	-0.44 (-0.93 to 0.06)
• > 49 years		reference	reference	reference		reference		reference		reference		reference
Speciality (1 = GP, 2 = not GP)	0.152	0.77 (-0.29 to 1.82)	0.756	-0.24 (-1.78 to 1.29)	0.281	-0.22 (-0.63 to 0.18)	0.568	-0.17 (-0.78 to 0.44)	0.841	0.04 (-0.04 to 0.42)	0.925	0.03 (-0.55 to 0.61)
Speciality experience												
• 0–5 years	0.863	-0.10 (-1.25 to 1.05)	0.415	-0.63 (-2.15 to 0.89)	0.550	-0.14 (-0.58 to 0.31)	0.499	-0.20 (-0.81 to 0.40)	0.896	-0.03 (-0.45 to 0.40)	0.410	0.24 (-0.34 to 0.82)
• 6–10 years	0.608	0.40(-1.13 to 1.93)	0.822	-0.19 (-1.88 to 1.49)	0.651	0.14 (-0.45 to 0.73)	0.862	-0.06 (-0.72 to 0.61)	0.799	0.07 (-0.49 to 0.63)	0.367	0.29 (-0.35 to 0.93)
• > 10 years		reference	reference	reference		Reference		reference		reference		reference
Full time/part time work (1 = full time, 2 = part time)	**0.021**	1.10 (0.17 to 2.04)	**0.020**	1.16 (0.19 to 2.12)	**0.031**	0.40 (0.04 to 0.77)	0.120	0.30 (-0.09 to 0.69)	0.633	-0.08(-0.43 to 0.26)	0.208	-0.24 (-0.62 to 0.13)
Private/ public clinic (1 = private, 2 = public)	0.186	-0.66 (-1.65 to 0.32)	0.094	-0.82 (-1.77 to 0.14)	**0.039**	-0.40 (-0.78 to -0.02)	**0.033**	-0.41 (-0.79 to -0.03)	0.231	0.22 (-0.14 to 0.57)	0.088	0.32 (-0.05 to 0.69)

*p−value < 0.05 is considered statistically significant

**Multivariate model is adjusted for gender, age, speciality, experience, full time/part time working, private/public clinic

### Confidence to treat obesity

While 99% of the physicians in our study (*n* = 232) felt confident to discuss lifestyle changes with their patients who suffered from obesity only 67% (*n* = 157) and 81% (*n* = 191) were confident to suggest medication for obesity or bariatric surgery, respectively (item 14).

The associations between feeling confident to suggest different obesity treatments and indices for knowledge, attitudes to obesity and adherence to obesity guidelines, using multiple linear regression, are presented in Table 4. There was a significant positive association between feeling confident to suggest medication (p < 0.001) or bariatric surgery (p = 0.002) with higher knowledge. Having positive attitudes to obesity was associated with higher confidence to suggest bariatric surgery to patients with obesity (p = 0.008).

**Table 4:** Multiple linear regression, the association between knowledge, attitude and adherence to obesity guidelines and confidence to treat obesity

	Knowledge		Attitude		Adherence	
	p-value^*^	B (95% CI)	p-value	B (95% CI)	p-value	B (95% CI)
I feel confident to discuss the following treatment options for obesity with my patients						
• Lifestyle changes	0.455	0.001(-0.001 to 0.003)	0.070	0.006 (< 0.001 to 0.012)	**0.007**	0.009 (0.002 to 0.015)
• Medication	**< 0.001**	0.037 (0.020 to 0.054)	0.559	0.013 (− 0.030 to 0.055)	0.548	0.014 (-0.32 to 0.059)
• Commercial obesity programs	0.347	0.008 (-0.009 to 0.026)	0.219	0.028 (-0.017 to 0.072)	0.174	-0.033 (-0.081 to 0.015)
• Bariatric surgery	**0.002**	0.023 (0.009 to 0.037)	**0.008**	0.048 (0.013 to 0.084)	0.562	0.011 (-0.027 to 0.049)

*p-value < 0.05

Adjusted R Square = 0.039

### Barriers to obesity treatment

**Fig. 3:**
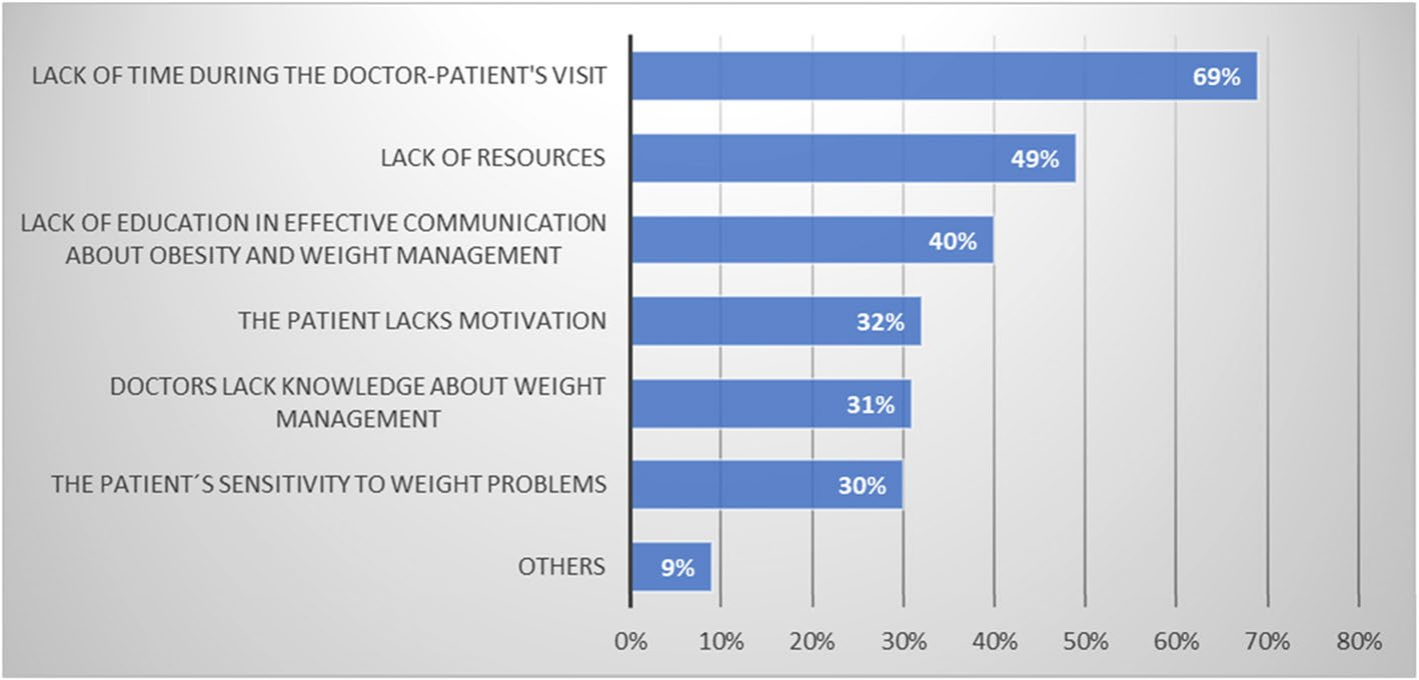
What is the biggest obstacle to discussing weight loss management with patients with obesity? (Item 30)

**Fig. 4:**
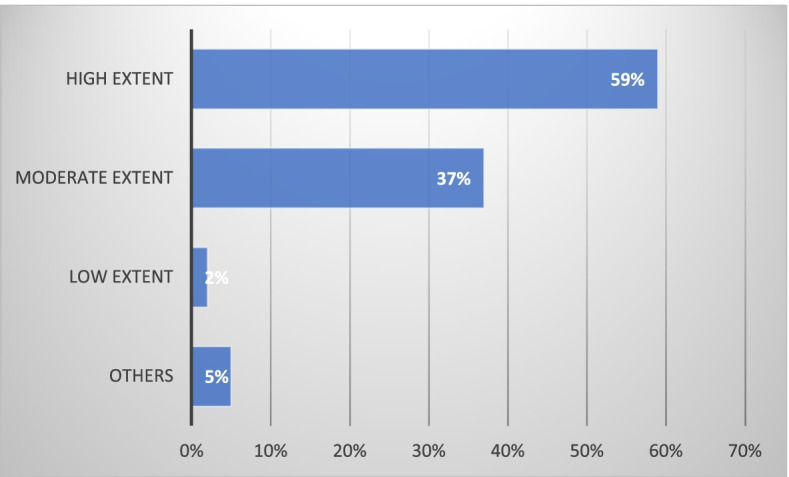
To what extent do your answers above actually reflect your clinical work in managing obesity? (Item 35)

Respondents perceived that the greatest barrier to managing patients with obesity was lack of time during physician/patient visits and lack of resources (item 30), Fig. 3. Almost a third of physicians (*n* = 72) also confessed that their knowledge about weight management was not sufficient. Consequently, only 59% of the respondents (*n* = 138) claimed that their responses to the items regarding adherence to guidelines actually reflected how they investigate, treat and follow-up patients with obesity (item 35), Fig. 4.

## Discussion

 The most important finding of this survey on obesity management by primary care physicians in Sweden was the positive association between physicians’ knowledge about obesity and better adherence to obesity guidelines. This result is in concordance with a similar survey study from the USA, with 148 physicians in a primary care setting, that found that physicians with greater knowledge and more positive attitudes toward obesity were more likely to provide weight management for their patients [19]. In our study, higher knowledge was associated with female gender, younger age (< 35 years) and working part-time. The association between younger age and higher knowledge might be due to younger physicians having more recent training and are more up to date on the guidelines. Physicians who work part-time might be engaged in other assignments such as teaching medical students or research and thus be more up to date. Our study also showed that respondents’ higher knowledge was associated with feeling more confident to suggest obesity medications or bariatric surgery to their patients. The result of our study also suggests that physicians with a positive attitude to obesity were more confident to discuss bariatric surgery with their patients who suffer from obesity.

In our study, BMI was used by all the primary care physicians to screen obesity. According to a systematic overview by Semlitsch et al. BMI was recommended for diagnosis and further assessment of obesity in 12 guidelines around the world [27].

Almost all primary care physicians in our study recommended regular physical activity and more than half of the physicians recommended smaller food portions to their patients with obesity. Almost all the physicians in our study felt confident to discuss lifestyle changes with their patients who suffered from obesity. According to most guidelines, lifestyle changes such as dietery modifications, physical activity, and behavioural interventions as well as multidisciplinary team therapy should be used to manage overweight and obesity as a long-term, chronic disease and the primary therapeutic goal should be improvement in the health of individuals by preventing or treating weight-related complications [27]. However, results of earlier studies indicate that lifestyle modifications such as diet and exercise are often ineffective strategies for sustained weight loss in patients with severe and complex obesity, and that the most effective weight loss programs resulted in a sustained weight loss of only 10% [28, 29]. Additionally, it is not completely evident whether this relatively small weight loss has a significant clinical impact on obesity comorbidities in individuals with severe and complex obesity [30, 31]. Only 67% and 81% of the respondents in our study felt confident to suggest medication for obesity or bariatric surgery, respectively. Yet, bariatric surgery has shown positive impacts on morbidity and mortality in individuals with severe and complex obesity with significant and sustained weight loss [32–34], and is considered to be cost effective for society and the healthcare system due to decreased costs related to treatment of obesity comorbidities [32, 35, 36].

This study shows that primary care physicians have an ambivalent attitude towards obesity. On one hand, the majority of physicians stated correctly that obesity is a chronic disease and due to several factors, such as genetics, changes in society, obesogenic medication and psychological disorders, which in turn result in changes in appetite regulation in the hypothalamus, i.e. feeling more hungry or late satiety. On the other hand, almost half of the physicians still perceived that lifestyle changes were the most effective weight reduction method for individuals with severe obesity and that obesity is due to lack of motivation, low self-control or laziness. Another paradox is that although almost all of the respondents in our study believed that physicians are able to help individuals with obesity to achieve a healthier weight, and more than half of physicians expressed that treatment of obesity should be prioritised, nevertheless a majority of the physicians (87%) stated that losing weight is primarily the patients’ responsibility. In another Swedish study it was shown that in 84% of cases it was the patient that initiated referral to bariatric surgery [15].

Swedish primary care physicians believed that the greatest obstacle to discuss weight loss management with their patients was lack of time during physician/patient consultations and lack of resources. However, an American study showed that lack of patient motivation was perceived to be the greatest barrier in managing obesity [19]. Respondents in other surveys indicated that the greatest barriers to weight management were lack of resources [37], lack of sufficient training [38], lack of time, scepticism about effectiveness of counseling and treatments, and patients’ lack of self-control [20, 38, 39].

In our study 20% of the respondents were unsure and 58% of the physicians did not believe that medication is an effective way to lose weight for individuals with moderate obesity. In a study from the USA from 2015, 30% of physicians expected a larger weight loss with pharmacotherapy than is realistic and 22% were unsure of the effects of pharmacotherapy for obesity [20].

### Implications

Primary care physicians might under-prioritise obesity treatment due to lack of knowledge and negative attitudes towards obesity. Future efforts focused on improving knowledge and attitudes about obesity and its treatment among primary care physicians and identification of barriers to choose the best obesity treatment could have an important effect on public health. Our results support the need for primary care physicians’ training about obesity issues to make them confident to adress obesity more often. Primary care physicians need more education on the efficacy and safety of obesity medications, especially when new agents become available. Furthermore, they need knowledge of indications, safety, and benefits of bariatric surgery as well as its pre- and postoperative care.

### Limitations and strengths

 To our knowledge, this is the first survey study exploring knowledge, attitudes, confidence to treat and adherence to obesity guidelines among primary care physicians in Sweden. The survey included case-vignettes, questions and items, which were formulated both negatively and positively in order to minimise the risk of an acquiescence response set. Some of the survey items were used in other international survey studies to make the items comparable. The items from other survey studies were translated from English to Swedish and back translated from Swedish to English by two independent researchers to make sure that the meaning of the items was not lost in translation. All the authors were fluent in both English and Swedish. The selection of four regions representing more than half the Swedish population was intended to give a good representation of the attitudes and knowledge of Swedish primary care physicians.

One limitation of the study is potential selection bias, i.e. those who responded to the questionnaire survey were perhaps more interested in obesity and its treatment. Thus, they could have higher knowledge, a more positive attitude to obesity, and better adherence to obesity guidelines than those who chose not to respond. Another limitation of the study is the use of case-vignettes instead of direct observation of the practice of physicians. However, the use of case vignettes has been shown to be a valid and reliable method that provides proxy data on physicians’ actual practice patterns [40]. One more limitation of this study is the low response rate of 14.3%. However, this response rate is similar to that of many other e-mail surveys [41] and could partially be explained by e-mail lists that were not completely updated since we received many automatic replies about invalid addresses. Another factor was the time pressure that many clinicians felt due to the COVID − 19 pandemic. Drop out analysis was not possible since the survey was anonymous.

## Conclusion

Healthcare systems around the world have been aware of the importance of treating obesity to prevent morbidity and premature mortality. However, the significance of treating obesity has been highlighted by the COVID-19 pandemic where obesity was an important risk factor for developing critical COVID-19 infection requiring intensive care [42].

 The most important finding of this study is the positive association between physicians’ knowledge and better adherence to obesity guidelines and feeling more confident to suggest obesity treatment. This study also shows that physicians had an ambivalent attitude towards obesity.

## Supplementary Information

**Additional file 1.** English translation of the questionnaire.

## Data Availability

The questionnaire is available as the “Supplementary Material” file. The datasets generated and analysed during the current study are not publicly available because survey respondents were not informed about data sharing and therefore did not give consent for this when they completed the survey. This was not yet customary at the time of this study. Data are available from the corresponding author on reasonable request.

## Abbreviations

BMI: Body mass index
EPM: Ethical Review Authority
WHO: World Health Organization
