# Supplementary material for: Primary care physicians’ knowledge and attitudes about obesity, adherence to treatment guidelines and association with confidence to treat obesity: a Swedish survey study
Source: BMC Prim Care. 2022 Aug 16;23:208. doi: 10.1186/s12875-022-01811-x (PMC9378264; doi:10.1186/s12875-022-01811-x)
Supplement: Supplementary file 1 — Additional file 1. English translation of the questionnaire. [file 12875_2022_1811_MOESM1_ESM.docx]

Appendix 1

English translation of the questionnaire

**Demographic information**

1. You are:
   - - Male
     - Female
2. Your age:

- <35
- 35-49
- >49

1. You are:

- Specialist in general practice
- Primary care physician without speciality in general practice

1. If you are specialist in general practice, how many years have you worked as a specialist?

- 0-5 years
- 6-10 years
- >10 years

1. You work clinically

- Full time
- Part time

1. You work at a

- Private primary health care
- Public primary health care

**Obesity items**

1. Physicians can help patients with obesity to attain a healthier weight.

- Yes
- No

1. Obesity is due to poor self-control.

- Yes
- No

1. Losing weight is primarily a patient’s responsibility.

- Yes
- No

1. Individuals with obesity lack motivation to lose weight.

- Yes
- No

1. I usually suggest obesity treatment to my patients, when I see them due to obesity related health problems.

- Yes
- No

1. Is obesity a priority to treat?

- Yes
- No

1. Obesity is a disease.

- Yes
- No

14. I feel confident discussing the following obesity treatment options with my patients:

- Lifestyle Changes
- Medications
- Commercial weight loss programs
- Obesity Surgery

1. Which of the following measurements do you use in your daily practice to assess obesity? (multiple response options possible.)

- BMI
- Waist circumference
- Waist-to-hip ratio
- Percentage of body fat (BF%)
- Weight
- Others (please mention here….)

1. Which lifestyle changes do you usually suggest to your patients with obesity? (multiple response options possible.)

- The plate model
- Smaller food portions
- Regular physical activity
- High fibre-foods
- Keyhole labelled products
- Nordic nutrition recommendations
- Cook from scratch, avoid whole-/semi-finished products
- Time restricted eating, 16 hours night fasting
- Cognitive behavioural therapy
- Behavioural changes
- Others (please mention here ……)

1. Which are the causes of obesity? (multiple response options possible.)

- Genetics
- Changes in the society (e. g. fast food industry, abundancy of cheap food)
- Socioeconomic status
- Lifestyle changes
- Pregnancy
- Obesogenic medication
- Intestine microbiota
- Lack of self-control
- Laziness
- Psychological disorders
- Others (Please mention here ….)

1. How is the appetite regulation disturbed for individuals with obesity?

- Constantly feeling hungry
- Late satiety
- Both above options
- I do not know

1. Hunger and satiety regulation sites are in:

- Brain cortex and can be controlled voluntarily and with good motivation
- Hypothalamus and is regulated by interactions between hormones and gastrointestinal organs.
- I do not know

1. Which of the following conditions might have an association with severe and complex obesity? (1= strongly disagree, 5=strongly agree)
2. Diabetes mellitus type II 1) 2) 3) 4) 5) 6) I do not know
3. Hypertension 1) 2) 3) 4) 5) 6) I do not know
4. Hyperlipidaemia 1) 2) 3) 4) 5) 6) I do not know
5. Sleep apnea 1) 2) 3) 4) 5) 6) I do not know
6. Gastroesophageal reflux disease (GERD) 1) 2) 3) 4) 5) 6) I do not know
7. Polycystic ovary syndrome (PCOS) 1) 2) 3) 4) 5) 6) I do not know
8. Female infertility 1) 2) 3) 4) 5) 6) I do not know
9. Joint pain 1) 2) 3) 4) 5) 6) I do not know
10. Obesity medications is an effective way to achieve weight loss in patients with moderate obesity.

- Yes
- No
- I do not know

1. Lifestyle changes are the most effective weight loss method for individuals with sever and complex obesity.

- Yes
- No
- I do not know

1. Approximately, what proportion of patients with severe obesity achieve a permanent 10% weight loss after 5 years with diet and behavioural changes?

- 10%-20%
- 30%
- 40%
- 60%
- I do not know

1. At what minimum BMI would you consider referring a patient WITHOUT obesity comorbidities to a dietician or specially trained nurse?

- BMI > 25
- BMI >28
- BMI >30
- BMI >35
- I do not know

1. At what minimum BMI would you consider referring a patient WITH obesity comorbidities to a dietician or specially trained nurse?

- BMI > 25
- BMI >28
- BMI >30
- BMI >35
- I do not know

1. At what minimum BMI would you consider prescribing obesity medication for a patient WITHOUT obesity comorbidities?

- BMI > 25
- BMI >28
- BMI >30
- BMI >35
- I do not know

1. At what minimum BMI would you consider prescribing obesity medication to a patient WITH obesity comorbidities?

- BMI > 25
- BMI >28
- BMI >30
- BMI >35
- I do not know

1. At what minimum BMI would you consider referring a patient WITHOUT obesity comorbidities to overweight clinic?

- BMI > 25
- BMI >28
- BMI >30
- BMI >35
- I do not know

1. At what minimum BMI would you consider referring a patient WITH obesity comorbidities to overweight clinic?

- BMI > 25
- BMI >28
- BMI >30
- BMI >35
- I do not know

1. What do you think is the biggest obstacle to discussing weight loss with obese patients? (Multiple response options possible)

- The patient lacks motivation
- Lack of time during clinical visits
- The patient's sensitivity to weight problems
- Lack of resources in primary care
- Lack of training in effective communication about obesity and weight management
- Doctors lack knowledge about weight management
- Other (please mention here …)

Case 1: A 32-year-old woman with a BMI of 36.0 kg/m^2^ seeks help because of infertility. She has irregular periods and increased hair growth under her chin.

31. What is the probability that you would discuss weight issues with this patient?

- Unlikely
- Small probability
- High probability

Case 2: A 25-year-old man with a BMI of 42.5 kg/m^2^ is seeking help for weight loss. He has made several attempts to lose weight by participating in Weight Watchers' programs and by using commercial powder diets. He has also been in contact with the dietician at your clinic. He has a long medical history of mild to moderate intermittent asthma and a 3-year medical history of hypertension. He is currently taking Enalapril 5 mg tablets and Amlodipin 10 mg tablets daily. Today's examination of the heart and lungs is normal.

32. The next step in treating this patient's obesity is to:

- Recommend lifestyle changes
- Refer to obesity surgery
- Refer to overweight clinic
- Treat with obesity medication
- I do not know
- Others (please mention here …)

33. How would you like to further assess this patient’s health: (Multiple response options possible)

- Lipid sampling
- Sampling for sugar or glucose tolerance
- Sleep registering
- No further investigation is needed
- I do not know
- Other comments (please mention here …)

Case 3: A 45-year-old man with a BMI of 38 kg/m^2^ and type II diabetes since five years ago. Although he uses Metformin 2000 mg and Repaglidine 16 mg daily, his HbA1C is 78 (9.3%). He has sleep apnoea and CPAP. But he does not use his CPAP ventilator because of the anxiety feelings he experiences when using it. The patient's father died due to a heart attack at age of 55 years.

34. The next step in treating the patient's obesity is to:

- Recommend lifestyle changes
- Refer to obesity surgery
- Refer to overweight clinic
- Treat with obesity medication
- I do not know
- Other comments (pleaser mention here …)

35. To what extent do you believe that your answers above actually reflect your clinical work on how to investigate, treat and follow your patient with obesity?

- Low extent
- Moderate extent
- High extent
- Other comments (pleaser mention here …)

Thank you for your participation
